# Synergistic Effects of Co on Nanoscale Dual-Precipitation in 2.3 GPa-Grade Steel

**DOI:** 10.3390/ma18132979

**Published:** 2025-06-23

**Authors:** Aijun Li, Jiaxin Liu, Yangxin Wang, Chundong Hu

**Affiliations:** 1 School of Materials Science and Engineering, Shanghai University, Shanghai 200444, China; liaijun001122@shu.edu.cn (A.L.); 1712042721@shu.edu.cn (J.L.); 2 Zhejiang Institute of Advanced Materials, Shanghai University, Jiaxing 314100, China

**Keywords:** Co, ultrahigh-strength steel, precipitation, diffusion coefficient

## Abstract

A novel ultrahigh-strength steel with Co and strengthened through nanoscale precipitation was developed. We found that the Co element had a synergistic effect on the precipitation process. The simulation results indicate that adding Co to steel can suppress the tracer diffusion coefficients of all the elements in the steel, hindering the atomic self-diffusion rate and long-range diffusion effect. A decrease in the atomic diffusion rate of precipitations will affect the nucleation, distribution, and growth of precipitations. The Atom probe tomography (APT) results indicate that the Co element not only dispersed uniformly in the matrix itself but also induced the uniform distribution of the precipitation phases. During the nucleation process of the precipitation, the rejected Co atoms formed small regions of high Co concentrations around the precipitation, inhibiting the coarsening of the precipitation. Under the synergistic effect of Co, the high number density of nanoscale NiAl and M_2_C enhanced the strength of the steel.

## 1. Introduction

Ultrahigh-strength steels (UHSSs) possess excellent strength, ductility, and toughness, making them widely used in critical aerospace applications such as bearings and aircraft landing gears [1,2,3]. UHSSs can be classified into low-alloy UHSSs, secondary hardening UHSSs, maraging steels, and precipitation hardening stainless UHSSs based on their different strengthening mechanisms. Low-alloy UHSSs are primarily strengthened by a quenched and low-temperature-tempered martensitic matrix along with finely dispersed carbides [4]. Secondary hardening UHSSs contain alloying elements such as Co, Ni, Mo, and Cr, with their strength mainly derived from the formation of fine, dispersed M_2_C precipitations and high-density dislocations in lath martensite during tempering [5]. Maraging steels achieve strengthening through the precipitation of intermetallic compounds, which is directly related to the types and amounts of alloying elements added [6]. Precipitation hardening stainless UHSSs are primarily based on maraging steels but incorporate a high Cr content to enhance their corrosion resistance [7]. 

As commercial demands for performance continue to rise, new ultrahigh-strength steels with higher strength and plasticity have been developed. Liu et al. [8] employed a cold-rolling process in the production of medium-Mn steel to obtain a thin, lamellar dual-phase structure of martensite and austenite, followed by carbon redistribution to improve the austenite stability, achieving both high strength and high fracture toughness. He [9] developed an ultrahigh-strength steel with a high dislocation density, where the microstructure was controlled via rolling to produce a metastable austenite embedded in a high-dislocation martensitic matrix, achieving a strength of 2200 MPa. Bhadeshia [10] utilized Si to suppress carbide precipitation in bainitic steel, leading to the development of super nano-bainitic steel.

The type and size of the precipitations in UHSSs play a crucial role in determining their mechanical properties [11]. Conventional UHSSs typically rely on a single type of precipitation for strengthening, such as M_2_C precipitations in secondary hardening steels or intermetallic precipitations (e.g., Ni_3_(Ti, Mo)) in maraging steels. However, these single-phase precipitations tend to nucleate preferentially at dislocations or defects, leading to an inhomogeneous distribution within the matrix, which limits the improvements in both the strength and ductility [12]. Therefore, a key research challenge lies in reducing the size of precipitations and promoting their uniform dispersion throughout the matrix.

The excellent mechanical properties of high-Co-Ni UHSSs (such as AF1410 and AerMet100) primarily rely on the strengthening effects of nanoscale precipitations, with their precipitation mechanisms involving complex interactions between alloying elements and phase transformation kinetics. In ferritic matrix (BCC) UHSSs, precipitation strengthening is mainly achieved through second-phase particles (e.g., carbides and intermetallic compounds) impeding dislocation motion, with its effectiveness significantly influenced by the size, distribution, and coherency of the precipitations.

During the development of secondary hardening UHSSs and maraging steels, cobalt (Co) is considered a key alloying element for enhancing their strength [13,14]. In recent years, the specific strengthening mechanisms of Co have been continuously explored. Co exhibits high mutual solubility with Fe in the crystal structure and primarily exists in steel as a substitutional solid solution. Due to the similar atomic radii of Co and Fe, the lattice distortion caused by the Co solid solution is limited, resulting in relatively weak solid solution strengthening [15,16]. Speich [17] found that in addition to its basic solid solution strengthening effect, Co also effectively delays dislocation recovery in the martensitic structure, promotes the fine and dispersed distribution of carbides, and enhances the secondary hardening peak. Perkas [18] reported that in maraging steels, Co reduces the solubility of Mo and W in the matrix, leading to a greater number of finer precipitations. Floreen [19] suggested that Co reduces the stacking fault energy of the body-centered cubic (BCC) lattice, increasing the dislocation density in the quenched state, which in turn provides more nucleation sites for precipitations. Gustafson used computational simulations to study the effect of Co on the coarsening of M_23_C_6_ carbides in P92 steel. The results showed that with the addition of 10% Co, the final average radius of the carbides after tempering at 600 °C for 30,000 h decreased by 30% [20,21].

At present, studies on the effect of Co on precipitations in UHSSs have mainly focused on individual precipitation types, such as M_2_C precipitations in secondary hardening steels [22,23] and intermetallic compounds like Ni_3_(Ti, Mo) in maraging steels [24]. These single-phase precipitations tend to nucleate preferentially at dislocations or defects and exhibit a non-uniform distribution in the matrix, which limits the improvements in the steel’s strength–ductility balance [12,25]. Although the macroscopic strengthening effect of Co in steels has been observed, its influence at the nanoscale, particularly in systems containing multiple coexisting nanoscale precipitations, remains unclear. In this study, a dual-precipitation-strengthened steel was designed. Through simulation and computational analysis, the precipitation characteristics of steels with and without Co were investigated. The steel was developed based on these findings, achieving a maximum ultimate tensile strength of 2.3 GPa. The synergistic role of Co in the dual-nanoprecipitation system was analyzed through the detailed characterization of NiAl and M_2_C.

## 2. Materials and Methods

**Figure 1:**
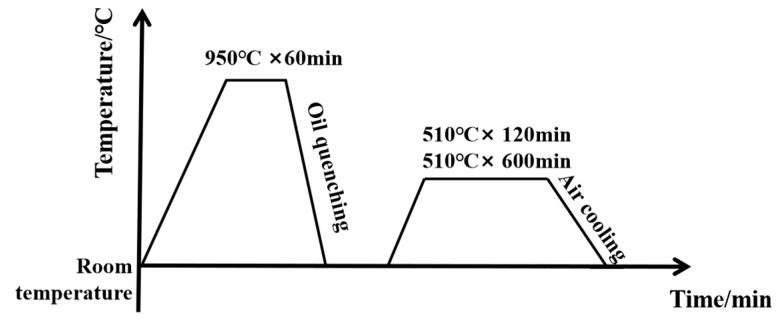
Schematic diagram of heat treatment process.

The steel had a nominal composition of Fe-0.26C-13.5Co-14.2Ni-1.2Al-2.1Cr- 1.8Mo-0.6W (wt.%) and was vacuum-induction-melted from a mixture of commercially pure metals (purity of > 99.9 wt.%). Then, it was vacuum-arc-remelted, homogenized at 1220 °C for 40 h, and then cast into a copper mold with a weight of 3 tons. The steel was subsequently forged into 8 mm × 1100 mm × 2000 mm blocks. As shown in Figure 1, the rolled plates were normalized at 950 °C for 1 h, followed by oil quenching, and then aged at 510 °C for separate periods of 120 min and 600 min. 

Tensile specimens were manufactured with a diameter of 3 mm and a gauge length of 15 mm. The tensile tests were performed using an INSTRON 5985 tensile testing machine (Instron, Shanghai, China) at a strain rate of 10^−4^ s^−1^. The elemental distribution at the atomic level was characterized by atom probe tomography (APT, LEAP 5000 XR) (Shanghai, China) conducted at a base temperature of 50 K, using high-voltage pulses with a pulse fraction of 0.2 and at a repetition of 125 kHz. APT samples were prepared through electrolytic polishing. Firstly, the sample was processed into a 0.5 mm × 15 mm square wire and then subjected to electrolytic polishing with a solution of 25% perchlorate acetic acid and 2% perchlorate ethylene glycol butyl ether to obtain a needle tip sample. The APT yield was approximately 2,000,000 atoms, with a volume of approximately 65,625 nm^3^. The APT data was reconstructed using the analysis software of Camera IVAS 3.6.8. The influence of the use of 13.5 wt.% Co-containing steel (the steel developed in this study) and Co-free steel (Fe-0.26C-14.2Ni-1.2Al-2.1Cr-1.8Mo-0.6W (wt.%)) on the diffusion of elements and the precipitation was calculated using the TCFE7 and MONFE2 modules in Thermo-Calc-PRISMA-2015a.

## 3. Results and Discussion

**Figure 2:**
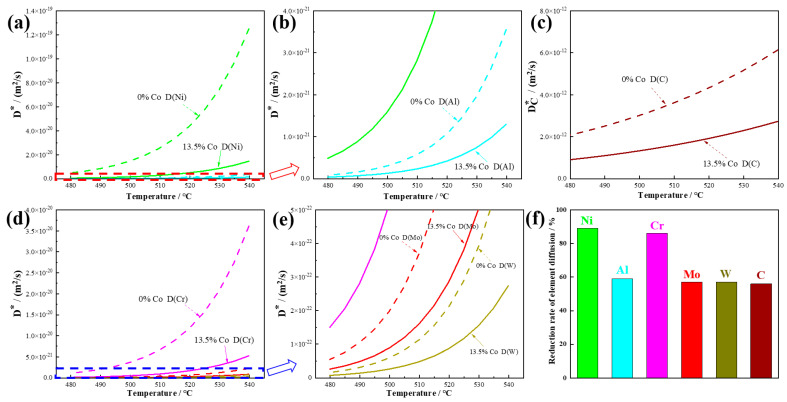
Tracer diffusion coefficients for elements in Co-free steel and 13.5% Co-containing steel. (**a**) Ni; (**b**) Al; (**c**) C; (**d**) Cr; (**e**) Mo and W; (**f**) reduction in element diffusion rate in Co-free steel and 13.5% Co-containing steel at 510 °C ((D_0% Co_ − D_13.5% Co_)/D_0% Co_).

The simulation of the tracer diffusion coefficients for Ni, Al, C, Cr, Mo, and W is plotted as a function of the temperature for Co-free steel and 13.5% Co-containing steel in Figure 2. It can be seen from Figure 2a–e that when 13.5% Co was added to the steel, the tracer diffusion coefficients of all the elements were significantly reduced. The difference in the tracer diffusion coefficients between Co-free steel and 13.5% Co-containing steel became larger and larger as the temperature increased. Figure 2f shows that 13.5% Co reduced the tracer diffusion coefficients of Al, Mo, W, and C in the matrix by almost 60% at a temperature of 510 °C. For Ni and Cr, the addition of 13.5% Co reduced their tracer diffusion coefficients by almost 95%. This indicates that the addition of Co to steel strongly suppressed the tracer diffusion coefficients of all the elements in the steel, hindering the self-diffusion rate and long-range diffusion effects of atoms. A decrease in the diffusion rate of precipitation atoms will affect the nucleation, distribution, and growth of precipitations [26,27].

To investigate the effect of Co on the precipitation behavior in steel, the TC-PRISMA precipitation module in Thermo-Calc was employed to simulate the size, distribution, and number density of MxC (x = 2 and 6) precipitations during aging at 510 °C for two alloy systems: a Co-free steel (Fe-0.26C-14.2Ni-1.2Al-2.1Cr-1.8Mo-0.6W, wt.%) and a 13.5 wt.% Co-containing steel (Fe-0.26C-13.5Co-14.2Ni-1.2Al-2.1Cr-1.8Mo-0.6W, wt.%). The TC-PRISMA module is based on the Langer–Schwartz theory and the Kampmann–Wagner numerical method, which are used to model the nucleation and growth of precipitations in multiphase systems under arbitrary heat treatment conditions. The growth behavior of the precipitations in the steel was analyzed using the classical CAJ model, and the calculations involved Equations (1)–(8), as shown below [28].

(1) $$\nu \left(c_{i}^{\frac{\beta }{\alpha }}-c_{i}^{\frac{\alpha }{\beta }}\right)=c_{i}^{\alpha /\beta }M_{i}\frac{\mu_{i}^{\alpha }-\mu_{i}^{\alpha /\beta }}{\xi_{i}r}$$

(2) $$\mu_{i}^{\beta /\alpha }=\mu_{i}^{\beta /\alpha }+\frac{2\sigma V_{m}^{\beta }}{r}$$

(3) $$\xi_{i}=\frac{\Omega_{i}}{2\lambda_{i}^{2}}$$

(4) $$\Omega_{i}=\frac{c_{i}^{\alpha }-c_{i}^{\alpha /\beta }}{c_{i}^{\beta /\alpha }-c_{i}^{\alpha /\beta }}$$

(5) $$2\lambda_{i}^{2}-2\lambda_{i}^{3}\sqrt{\pi }\exp\left(\lambda_{i}^{2}\right)\mathrm{erfc}\left(\lambda_{i}\right)=\Omega_{i}$$

(6) $$\nu =\frac{K}{r}[\Delta G_{m}-\frac{2\sigma V_{m}^{\beta }}{r}]$$

(7) $$K={\left[\sum_{i}\frac{{\left(X_{i}^{\frac{\beta }{\alpha }}\left(r\right)-X_{i}^{\frac{\alpha }{\beta }}\left(r\right)\right)}^{2}\xi_{i}}{X_{i}^{\frac{\alpha }{\beta }}\left(r\right)M_{i}}\right]}^{-1}$$

(8) $$\nu^{\prime }=\nu (1+r\sqrt{4\pi N_{V}\langle r\rangle })$$

In Equation (1), *υ* represents the interface velocity, and *n* is the number of components. *C* denotes the volume concentration of components at the interface between the matrix and the precipitation; *M* is the mobility of the atoms in the matrix phase; and μ refers to the chemical potential at the interface. ζ is the effective diffusion distance factor in Equation (3). *Ω* denotes the dimensionless supersaturation in Equation (4). *ΔGm* is the driving force for precipitation in Equation (6). *Xi(r)* represents the composition along the tie-line in the matrix phase in Equation (7). In Equation (8), *<r>* is the average equivalent radius of the precipitations, and *Nv* is the number density of the precipitations.

**Figure 3:**
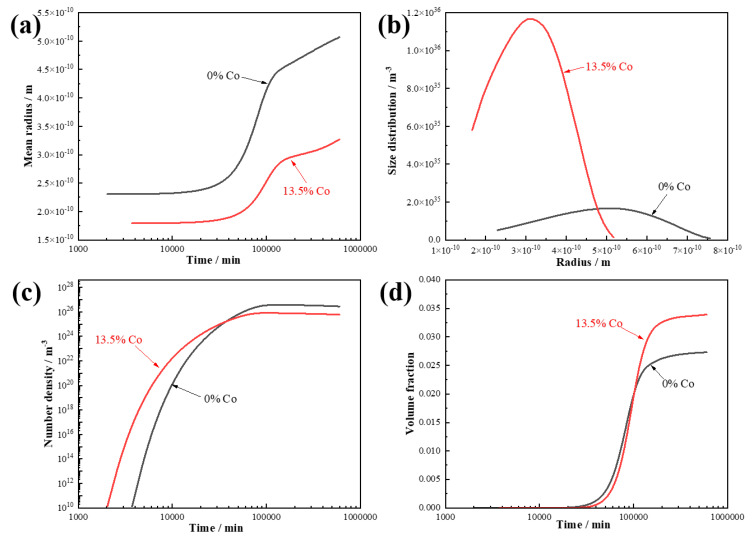
Characteristics of carbides (MxC, x = 2 and 6) in Co-free steel and Co-containing steel at 510 °C. (**a**) Mean radius; (**b**) size distribution; (**c**) number density; (**d**) volume fraction.

The simulation results for the carbides in the steel are shown in Figure 3 [29]. Figure 3a presents the relationship between the aging time and the equivalent radius of the carbides for both steels. It can be observed that the carbides in the 13.5 wt.% Co steel had a smaller equivalent radius, and the onset of significant coarsening was delayed. Figure 3b illustrates the size distribution of the carbides in the two steels. The results indicate that the addition of 13.5 wt.% Co significantly refined the size distribution of the carbides. The evolution of the number density and volume fraction of the carbides with the aging time are shown in Figure 3c,d. Compared to the Co-free steel, the 13.5 wt.% Co steel exhibited a higher density of carbides when the ageing time were less than 10,000 min. After 600,000 min of aging, the volume fraction of the carbides in the Co-containing steel was 1.2 times that in the Co-free steel.

Overall, the simulation results suggest that the addition of 13.5 wt.% Co can reduce the carbide size, improve the size distribution, increase the number density, and enhance the volume fraction. Although the computational approach can predict the precipitation trends of carbides, it is typically based on models of relatively large precipitations, making it difficult to accurately reflect the behavior of nanoscale precipitations in actual steels. Therefore, while the simulations provide useful guidance for alloy design, discrepancies still exist between the predicted results and the actual microstructure. Moreover, the simulations primarily captured changes in the size, number density, and volume of precipitations after approximately 1000 min of aging. In real aging processes, local variations in the Co concentration across different regions of the steel may also influence the precipitation behavior.

**Figure 4:**
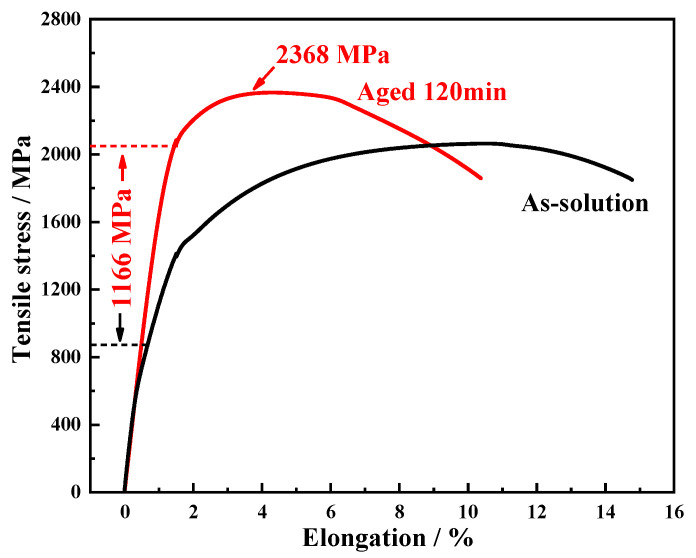
Engineering stress–strain curves of the developed steel in solution-treated (annealed at 950 °C for 60 min) and aged (aged at 510 °C for 120 min) conditions.

Following the above simulations and calculations, a steel alloy containing 13.5 wt.% Co was fabricated to investigate the influence of Co on nanoscale precipitations and its strengthening effect. Tensile tests were conducted under different heat treatment conditions, and the resulting engineering stress–strain curves are shown in Figure 4. In the solution-treated condition, the ultimate tensile strength (UTS) was 2054 ± 5 MPa, with a total elongation of 13.5 ± 0.7%. After an aging treatment, the UTS increased to 2368 ± 3 MPa; however, the total elongation decreased to 10.4 ± 0.6%. Moreover, a significant increase in the yield strength—by 1166 MPa—was observed after the aging treatment. These results indicate that under the Co-containing condition, the nanoscale precipitations exhibited a strong hardening effect.

**Figure 5:**
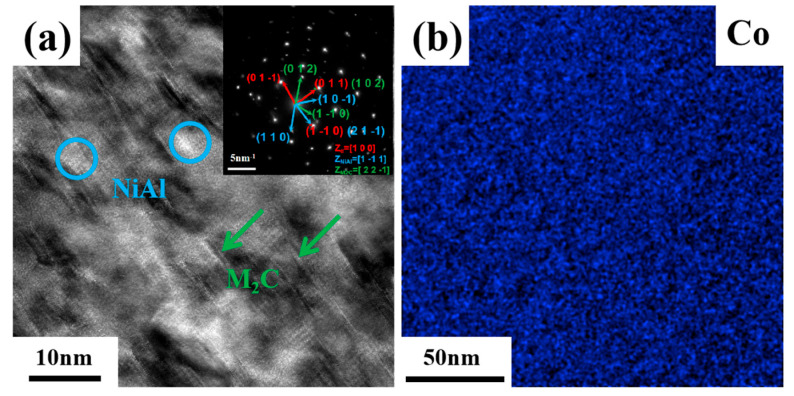
Microstructural characteristics and elemental information. (**a**) High-resolution TEM image of the sample; (**b**) EDS maps of distribution of Co.

**Figure 6:**
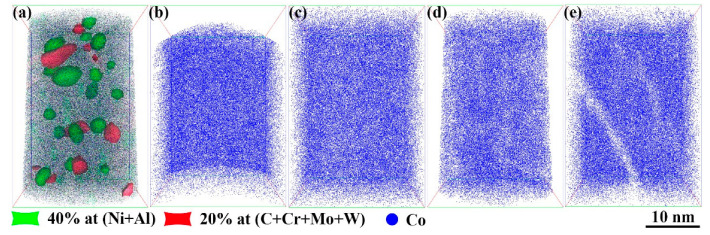
Atomic distribution of precipitations and Co in steel aged at 510 °C for different durations: (**a**) aging for 120 min; (**b**) solid solution state; (**c**) aging for 30 min; (**d**) aging for 120 min; (**e**) aging for 600 min.

Figure 5 illustrates TEM images of the sample aged at 510 °C for 120 min. Two kinds of NiAl and M_2_C precipitations were uniformly distributed in the martensitic matrix. As shown in Figure 5b, the Co element was uniformly distributed in the martensitic matrix. The upper-right corner of Figure 5a shows the corresponding Fast Fourier Transform (FFT) pattern. The red arrow along the [100]-zone axis shows the diffraction points of martensite. The blue arrow along the [1-11]-zone axis identifies the diffraction points of NiAl, while the green arrow along the [22-1]-zone axis corresponds to the diffraction points of M_2_C. Figure 6 presents a three-dimensional reconstruction of the precipitations in the steel and the distribution of the Co atoms under different aging conditions. Figure 6a shows a reconstructed image of nanoscale NiAl and M_2_C after 2 h of aging treatment. The fine, spherical, and closely packed precipitations were identified as NiAl, defined by iso-concentration surfaces of 40 at.% for both Ni and Al. The precipitations rich in C, Cr, Mo, and W were identified as M_2_C, made up of iso-concentration surfaces of 20 at.% for C, Cr, Mo, and W. The number density of the NiAl precipitations was approximately 1.35 × 10^24^ m^−3^, with sizes ranging from 1 to 8 nm. The number density of the M_2_C precipitations was about 0.83 × 10^24^ m^−3^, with lengths between 2 and 10 nm. The NiAl precipitations, typically spherical and small (approximately 2–5 nm in diameter), were uniformly distributed within the matrix, with a number density of about 0.9 × 10^24^ m^−3^. The M_2_C precipitations, mainly short, rod-like structures (approximately 2–3 nm in diameter and 2–5 nm in length), were primarily located around the NiAl precipitations, with a number density of approximately 0.5 × 10^24^ m^−3^. The M_2_C precipitations were often connected at their interfaces to adjacent NiAl particles, indicating that the NiAl phase influenced the nucleation sites and mechanisms of the M_2_C precipitations. Figure 6b–e show the distribution of the Co atoms in the solution-treated state and after aging for 30 min, 120 min, and 600 min, respectively. In the solution-treated state, the Co atoms were uniformly distributed. After 30 min of aging, no significant change in the Co distribution was observed. However, by 2 h, localized low-concentration regions of Co began to appear, and by 10 h, these low-concentration zones became more pronounced. These areas corresponded to regions occupied by rod-like precipitations, indicating that Co atoms were expelled into the matrix as the precipitations grew. These results demonstrate that although Co was not a primary constituent of the precipitations, it played a critical role in the nucleation and growth of NiAl and M_2_C.

**Figure 7:**
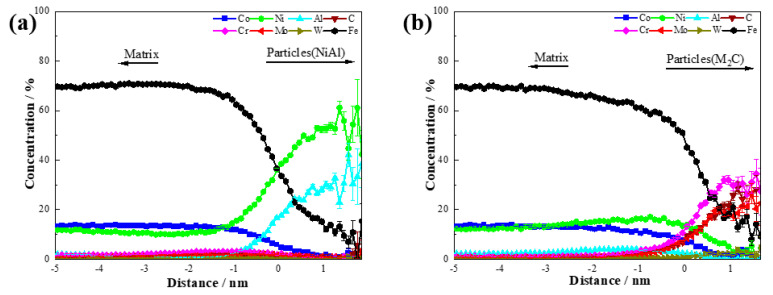
Concentration distribution of precipitations: (**a**) NiAl; (**b**) M_2_C.

The concentration distributions of NiAl and M_2_C in the steel after aging at 510 °C for 120 min are shown in Figure 7. The core composition of the NiAl precipitations was approximately 61.1 ± 11.5 at.% Ni, 33.3 ± 11.1 at.% Al, and 0 at.% Co. For M_2_C, the core composition consisted of 27.3 ± 9.5 at.% Cr, 22.7 ± 8.9 at.% Mo, 4.5 ± 4.4 at.% W, 27.3 ± 9.5 at.% C, and 0 at.% Co. These results indicate that during the nucleation process of NiAl and M_2_C, Co atoms were excluded from the precipitations. Although Co did not enter the precipitations, it played a crucial role in the nucleation and growth of both NiAl and M_2_C as a non-precipitating element.

**Figure 8:**
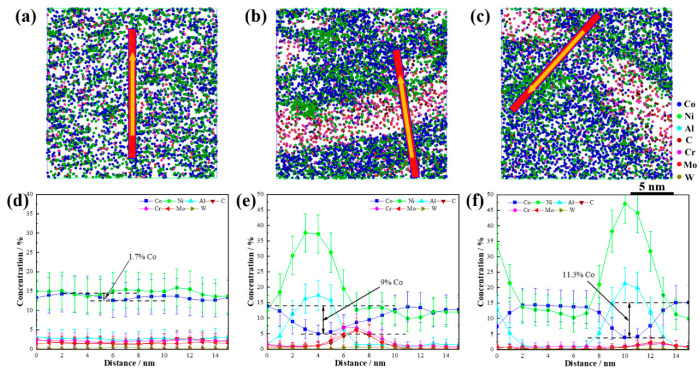
Atomic distribution map and concentration profile visualization: (**a**,**d**) solution-treated; (**b**,**e**) aged at 510 °C for 120 min; (**c**,**f**) aged at 510 °C for 600 min.

To investigate the synergistic effect of Co on the dual nanoscale precipitations, cross-sectional analyses of the precipitations at various aging stages were performed, as shown in Figure 8. In the solution-treated state, the Co concentration fluctuations were minimal, with an average value of approximately 12.9% and a maximum difference between the high- and low-concentration areas of only 1.7%. After aging for 120 min, the Co content in the core of the NiAl precipitations decreased to 5%, and in the M_2_C precipitations it decreased to 8.5%. The highest Co concentration at the phase boundary was 13.9%. These results show that with an increasing aging time, the Co atoms were gradually expelled from the precipitations into the matrix. The maximum Co concentration difference between the precipitations and the matrix was 9%. After 600 min of aging treatment, the precipitations began to coarsen slightly yet still maintained their nanoscale size. At this stage, the Co content in the NiAl core further decreased to 3.8%, and it decreased to 7.6% in M_2_C. The highest Co concentration at the boundary of the precipitations was 15.2%, resulting in a maximum Co concentration difference of 11.3% between the precipitations and the matrix.

Based on the simulation results, it can be concluded that Co suppressed the diffusion of the solute atoms that formed the precipitations, promoting the short-range segregation of these atoms in the matrix. This effect induced the uniform nucleation of the precipitations and improved their distribution and number density [30,31]. As the precipitations nucleated and grew, Co atoms were gradually released into the surrounding matrix. The regions of high Co concentrations around the precipitations hindered the long-range diffusion of atoms away from the precipitation–matrix interface, thereby retarding precipitation coarsening [32]. Even after long-term aging treatment, the precipitations maintained a high number density and nanoscale size. Thus, Co exhibited a synergistic strengthening effect in the newly designed steel.

## 4. Conclusions

A new type of cobalt containing ultrahigh-strength steels (UHSSs) was designed and produced. From simulation and APT results, it was verified that Co atoms play a synergistic role in promoting the uniform nucleation of precipitations and inhibiting their growth in steel. During the aging process, the diffusion rate of Al and Ni atoms is fast, and the NiAl phase has a low mismatch with the matrix, resulting in low interfacial energy and a low nucleation barrier. Under the action of Co, the NiAl phase can quickly and uniformly precipitate into small particles. When NiAl precipitates, the lattice distortion energy generated at the phase’s interface with the matrix provides nucleation energy for M_2_C, promoting the uniform and fine precipitation of M_2_C. With an increase in the aging time, NiAl and M_2_C can mutually inhibit each other’s growth, keeping the precipitations in the steel in a small and dispersed state and improving the mechanical properties of the steel. This new type of steel has ultrahigh strength and good plasticity. Its ultimate tensile strength is 2368 ± 3 MPa, and its total elongation is 10.4 ± 0.6%.

## Data Availability

The original contributions presented in this study are included in the article. Further inquiries can be directed to the corresponding authors.
